# Complete genome sequence of *Staphylococcus haemolyticus* UMB6531B, isolated from a perineal swab

**DOI:** 10.1128/mra.00371-25

**Published:** 2025-07-08

**Authors:** Alex Kula, Olive Kolar, Karolina Gilewicz, Zainab Qureshi, Anmol Singh, Alan J. Wolfe, Catherine Putonti

**Affiliations:** 1 Department of Biology, Loyola University Chicago 2456, Chicago, Illinois, USA; 2 Bioinformatics Program, Loyola University Chicago 2456, Chicago, Illinois, USA; 3 Department of Microbiology and Immunology, Stritch School of Medicine, Loyola University Chicago 550858, Maywood, Illinois, USA; University of Maryland School of Medicine, Baltimore, Maryland, USA

**Keywords:** *Staphylococcus haemolyticus*, overactive bladder symptoms, urogenital microbiome

## Abstract

*Staphylococcus haemolyticus* UMB6531B was isolated from a perineal swab of a female with overactive bladder symptoms and sequenced to better characterize opportunistic pathogens of the female urogenital tract. Here, we present the complete genome sequence, which includes the chromosome as well as two complete plasmid sequences.

## ANNOUNCEMENT

Although *Staphylococcus haemolyticus* is commonly found in the human skin microbiota (1), it is an opportunistic pathogen in other anatomical sites (2–4). In our continued effort to sequence microbes of the female urogenital microbiota, here we present the complete genome sequence of a *S. haemolyticus* isolate from a perineal swab collected from a female participant with overactive bladder symptoms as part of a prior study (5).

*S. haemolyticus* UMB6531B was isolated using the expanded quantitative urinary culture protocol (6) as part of a prior institutional review board (IRB) approved study (Loyola University Chicago, IRB nos. LUC 207152 and 207777). We obtained this strain, streaked on a Columbia nalidixic acid agar plate incubated at 35°C with 5% CO_2_ for 24 hours, from the Loyola Urinary Education and Research Collaborative (LUEREC) collection. A single colony was picked to inoculate 1 mL of brain heart infusion (BHI) media, which was then incubated under the same culture conditions. The liquid culture was then stuck on a BHI plate and incubated under the same conditions prior to sending it out for sequencing at SeqCenter (Pittsburgh, PA, USA). SeqCenter performed DNA extraction (ZymoBIOMICS DNA Miniprep Kit, per the manufacturer’s protocol) and prepared both an Illumina and Nanopore library using the DNA Prep kit (Illumina) with custom IDT 10 bp unique dual indices with a target size of 280 bp and the PCR-free Oxford Nanopore Technologies Ligation Sequencing Kit (SQK-NBD114.24) with the NEBNext Companion Module (E7180L), respectively (no size selection or fragmentation was performed for the ONT library). Sequencing was performed using an Illumina NovaSeq X Plus and an Oxford Nanopore MinION Mk1B sequencer or a GridION sequencer using R10.4.1 flow cells. Demultiplexing, quality control, and adapter trimming of the Illumina reads were performed by SeqCenter with bcl-convert (v4.2.4). Guppy (v6.5.7) was used for super-accurate basecalling, demultiplexing, and adapter removal for the Nanopore reads. In total, Illumina sequencing produced 6,367,606 reads (2 × 151 bp) and Nanopore sequencing produced 71,830 reads (N50 = 5,889 bp).

Hybrid assemblies were performed using the web tool BV-BRC (v3.36.16.5) (7) with the “auto” option, which trimmed reads via Trim Galore (v0.6.5dev; https://github.com/FelixKrueger/TrimGalore) and normalized reads using BBNorm (vOctober 19, 2017; https://sourceforge.net/projects/bbmap/). The filtered reads were assembled with Flye v2.9.1-b1780 (8) and then polished by two successive rounds of Racon v1.4.20 (9) and Pilon v1.23 (10). The average long read coverage was 130.45×, and the average short read coverage was 140.88×. The genome assembly was annotated upon its deposit to NCBI via PGAP v6.9 (11). Default parameters were used for all software tools.

Bandage (v0.8.1) (12) showed that the assembly consisted of three circular sequences, totaling 2,322,835 bp with a GC content of 32.85% and N50 score of 2,315,435 bp. Circularization and rotation of the chromosome to *dnaA* was conducted as part of the BV-BRC pipeline. The longest circular contig, 2,315,435 bp, represents the complete chromosome sequence, while the two smaller contigs are plasmids: pUMB6531B_1 (4,315 bp) and pUMB6531B_2 (3,085 bp). Further sequencing of isolates from the female urogenital tract provides greater insight into the microbial communities associated with health and symptoms.

## Data Availability

Both Illumina and Nanopore raw reads have been deposited in NCBI and are available through the SRA database, accession nos. SRR32614270 and SRR32614271, respectively. Assemblies have been deposited in GenBank under the accession nos. CP185994.1 (chromosome), CP185995.1 (pUMB6531B_1), and CP185996.1 (pUMB6531B_2).
